# Supplementary figures and images for: Effect of method of deduplication on estimation of differential gene expression using RNA-seq
Source: PeerJ. 2017 Mar 16;5:e3091. doi: 10.7717/peerj.3091 (PMC5357343; doi:10.7717/peerj.3091)

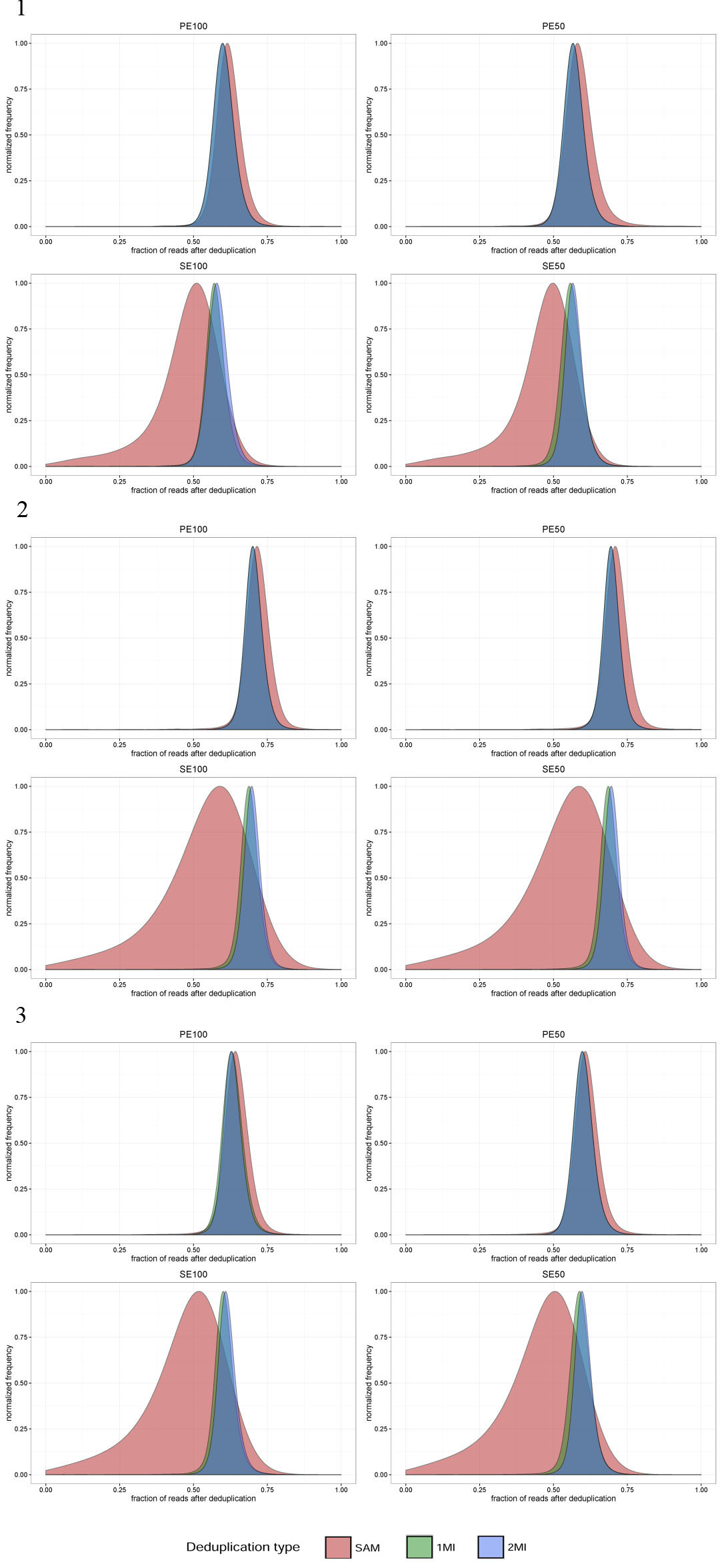

Supplement: Figure S1 — Color legend is the same as in Fig. 1. [file peerj-05-3091-s005.jpg]

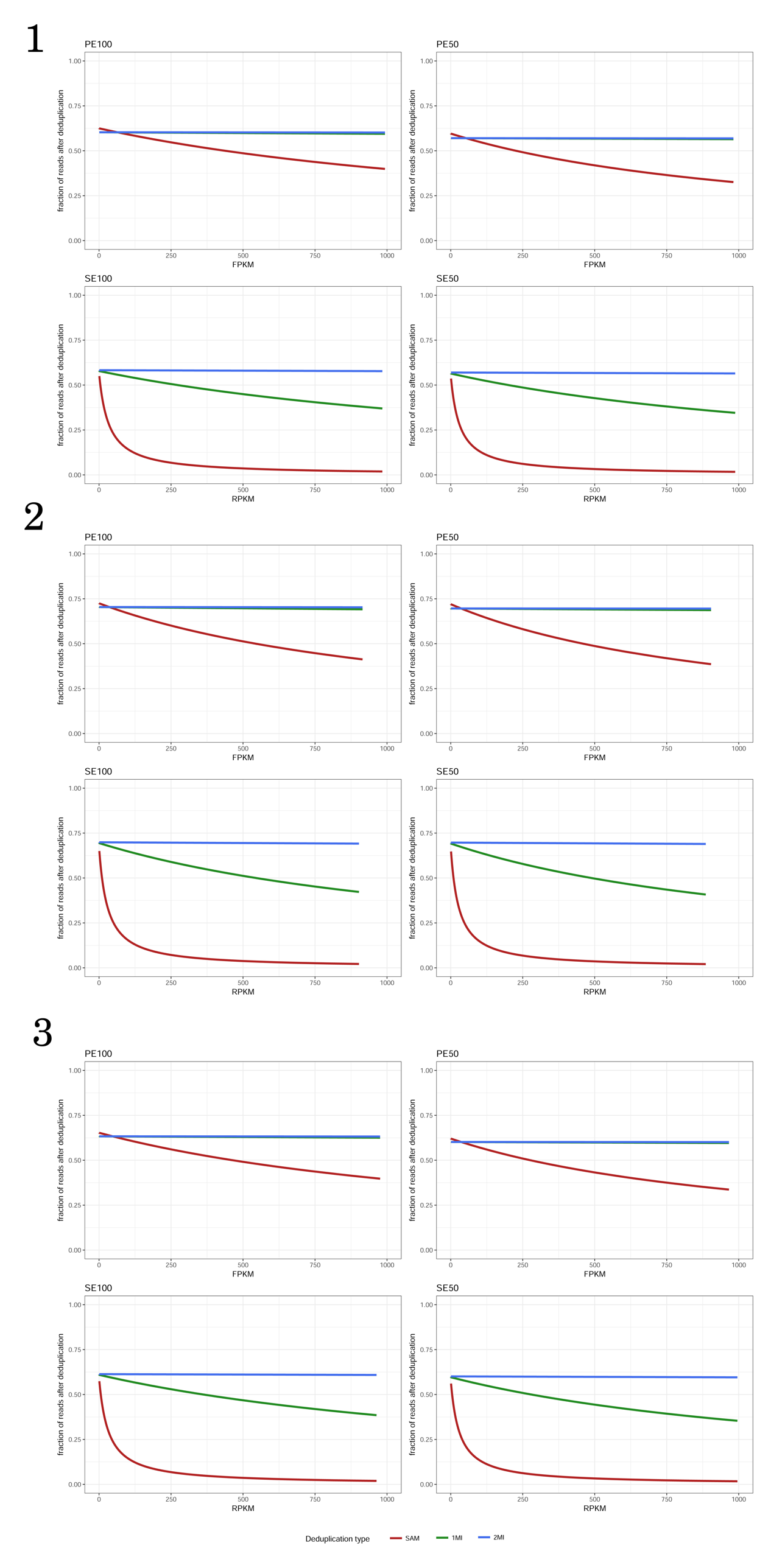

Supplement: Figure S2 — Color legend is the same as in Fig. 2. [file peerj-05-3091-s006.jpg]
